# Supplementary material for: Formyl peptide receptor 2 determines sex-specific differences in the progression of nonalcoholic fatty liver disease and steatohepatitis
Source: Nat Commun. 2022 Jan 31;13:578. doi: 10.1038/s41467-022-28138-6 (PMC8803937; doi:10.1038/s41467-022-28138-6)
Supplement: Supplementary file 1 — Supplementary Information [file 41467_2022_28138_MOESM1_ESM.pdf]

Formyl peptide receptor 2 determines sex-specific differences in the progression of nonalcoholic fatty liver disease and steatohepatitis

Chanbin Lee[1], Jieun Kim[1], Jinsol Han[1], Dayoung Oh[1], Minju Kim[1], Hayeong Jeong[1], Tae-Jin Kim[1,2], Sang-Woo Kim[1,2], Jeong Nam Kim[1,3], Young-Su Seo[1,3], Ayako Suzuki[4], Jae Ho Kim[5] and Youngmi Jung[1,2*]

[1]Department of Integrated Biological Science, [2]Department of Biological Sciences, [3]Department of Microbiology, College of Natural Science, Pusan National University, Pusan, 46241, Republic of Korea

[4]Division of Gastroenterology and Hepatology, Duke University, Durham, North Carolina

[5]Department of Physiology, Pusan National University School of Medicine, Pusan National University, Yangsan, 50612, Republic of Korea

Supplementary Figures

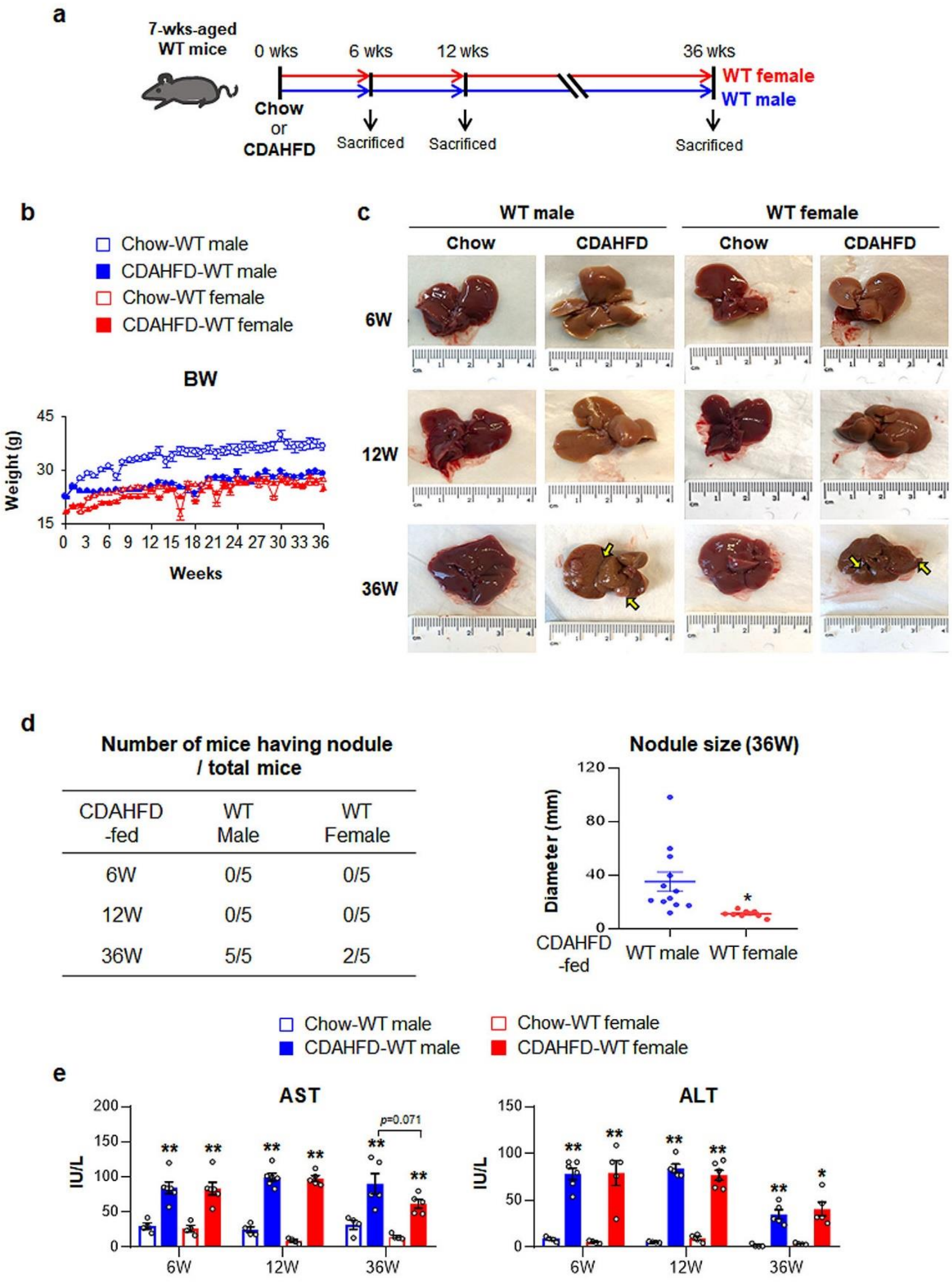

Supplementary Figure 1. CDAHFD generates the experimental animal model of human-like NAFLD

(a) A scheme for animal experiment mimicking human-like NAFLD. 7-week-old WT male and female mice were fed either normal chow (Chow-WT male and Chow-WT female) or CDAHFD (CDAHFD-WT male and CDAHFD-WT female) for 6, 12 and 36 weeks. (b) body weight of these mice for 36 weeks. Data represent the mean ± S.E.M. (c) Representative macroscopic appearance of livers from each group. Liver image of CDAHFD-female mouse having nodules is shown. Yellow arrows indicate nodules. (d) The number of mice having nodule was counted and diameter of nodules from these mice was measured and plotted. Data represent the mean ± S.E.M. (*p<0.05, **p<0.005 vs CDAHFD-WT male). (e) Levels of serum AST and ALT in all WT mice. Data represent the mean ± S.E.M. (n ≥ 3 /group, *p<0.05, **p<0.005 vs own control). Gray circles represent individual data points. See Supplementary Data for statistical details.

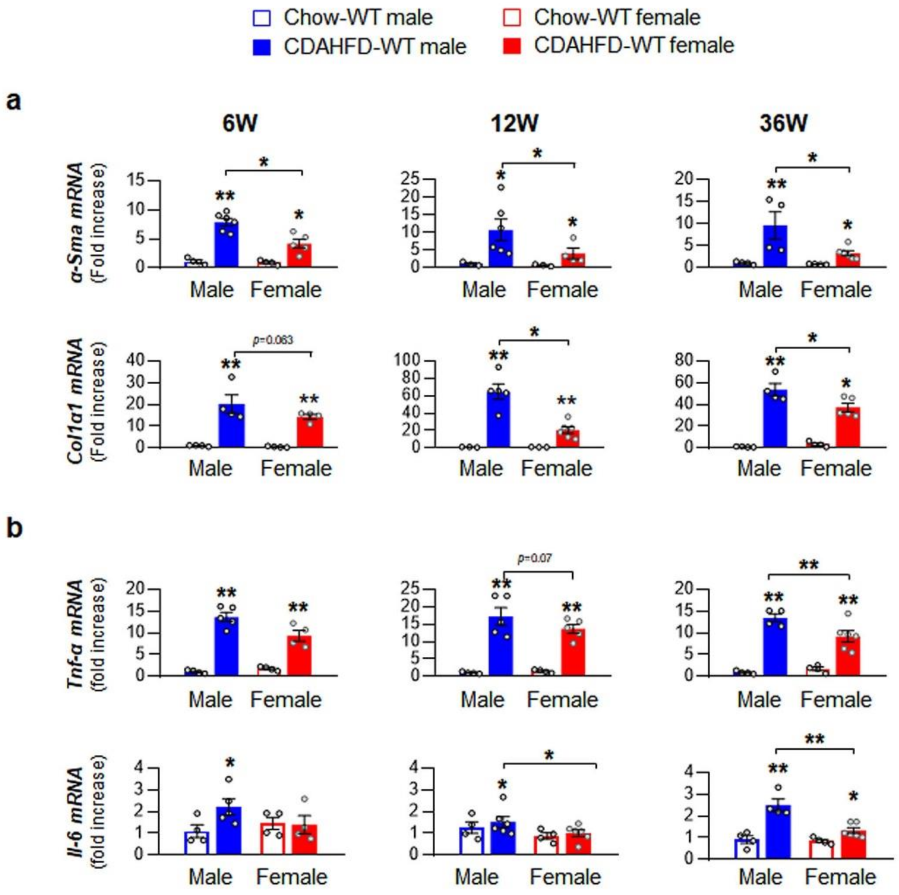

Supplementary Figure 2. Upregulation of fibrotic and pro-inflammatory markers in CDAHFD-treated mice

(a) qRT-PCR analysis of fibrotic markers, *α-Sma, Col1α1,* and (b) inflammatory markers including *Tnf-α, Il-6* in WT male and female mice fed either chow or CDAHFD for 6, 12 and 36 weeks. Data represent the mean ± S.E.M. (n ≥ 3 /group, **p*<0.05, ***p*<0.005 vs own control). Gray circles represent individual data points. See Supplementary Data for statistical details.

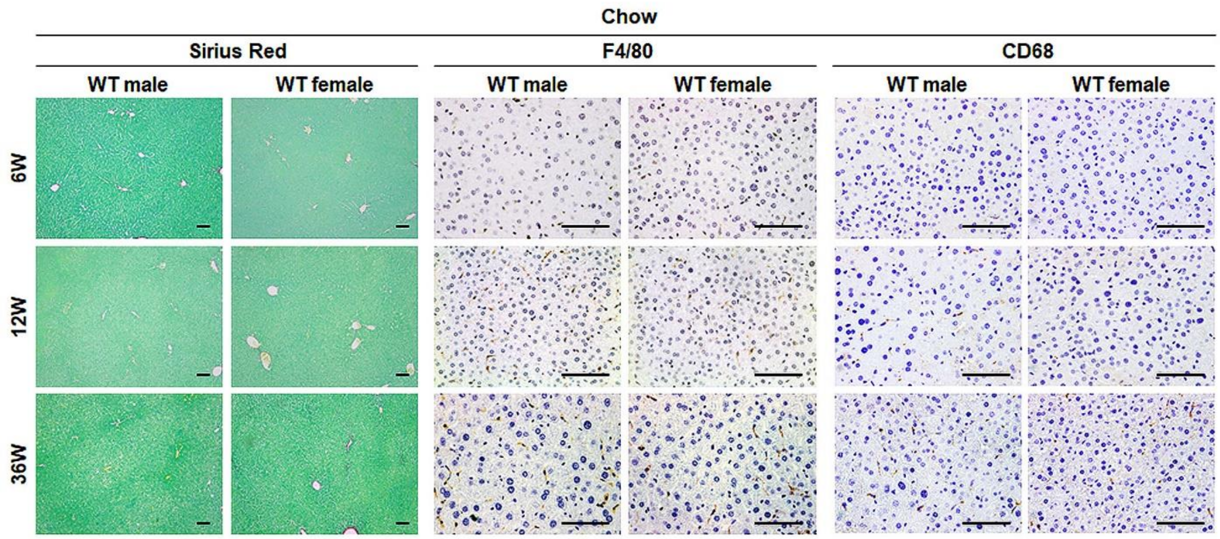

Supplementary Figure 3. Hepatic fibrosis and inflammation are absent in chow-fed mice
Representative images of Sirius red- (left panel, n=4) or F4/80- (middle panel, n=3) or CD68-stained (right panel, n=4) liver sections from the Chow-WT groups (Scale bar, 50μm).

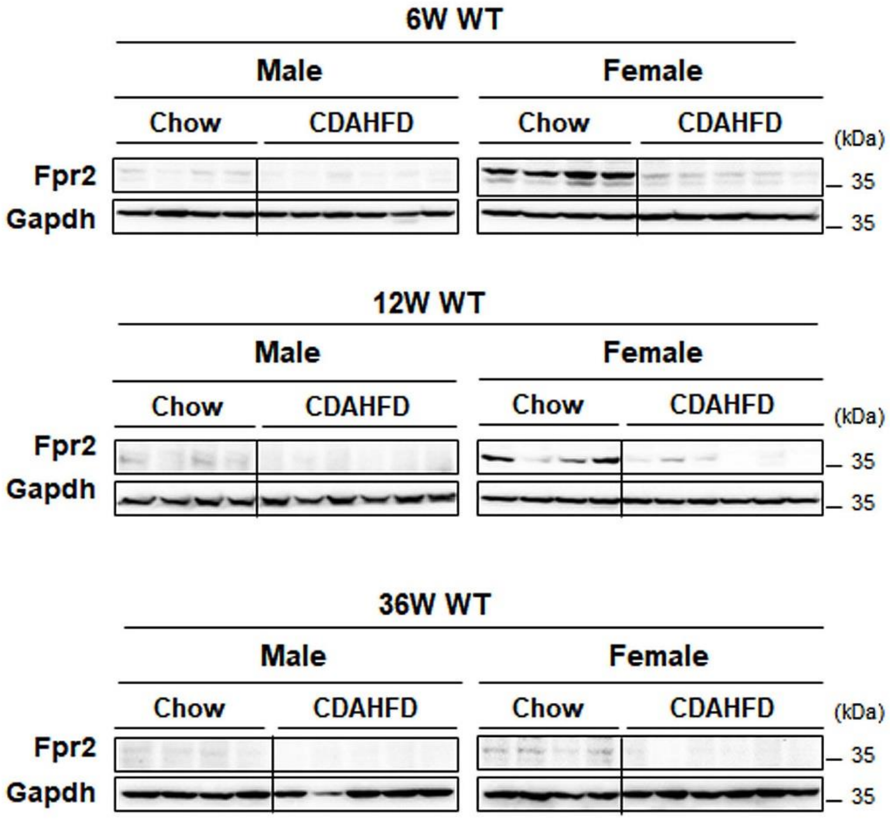

Supplementary Figure 4. Hepatic expression of FPR2 protein chow- or CDAHFD- fed WT mice

Western blot analyses for hepatic Fpr2 from WT male and female mice fed either chow or CDAHFD. Gapdh was used as internal control. Data shown represent one of three experiments with similar results.

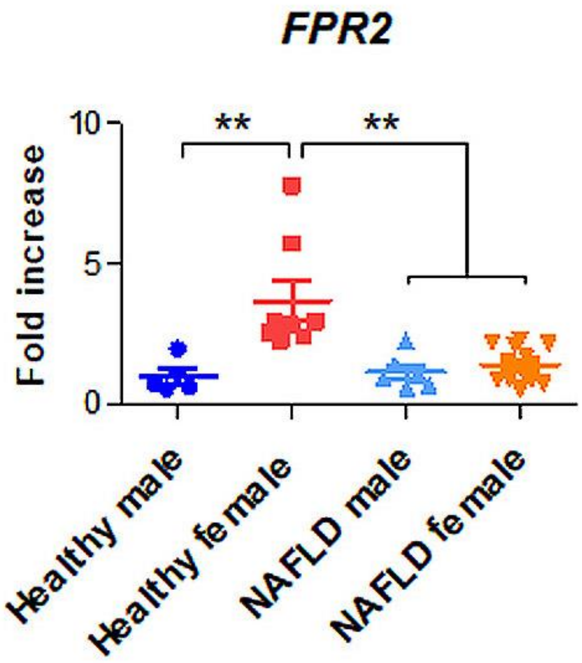

Supplementary Figure 5. Microarray analysis of FPR2 in adolescent peoples with or without NAFLD

Microarray analysis for hepatic *FPR2* from healthy and patients with NAFLD at age range from 13-19 (GSE66676) was displayed as the relative expression for own healthy male. Data represent the mean ± S.E.M (n ≥ 5 /group, *p<0.05, **p<0.005 vs healthy male). See Supplementary Data for statistical details.

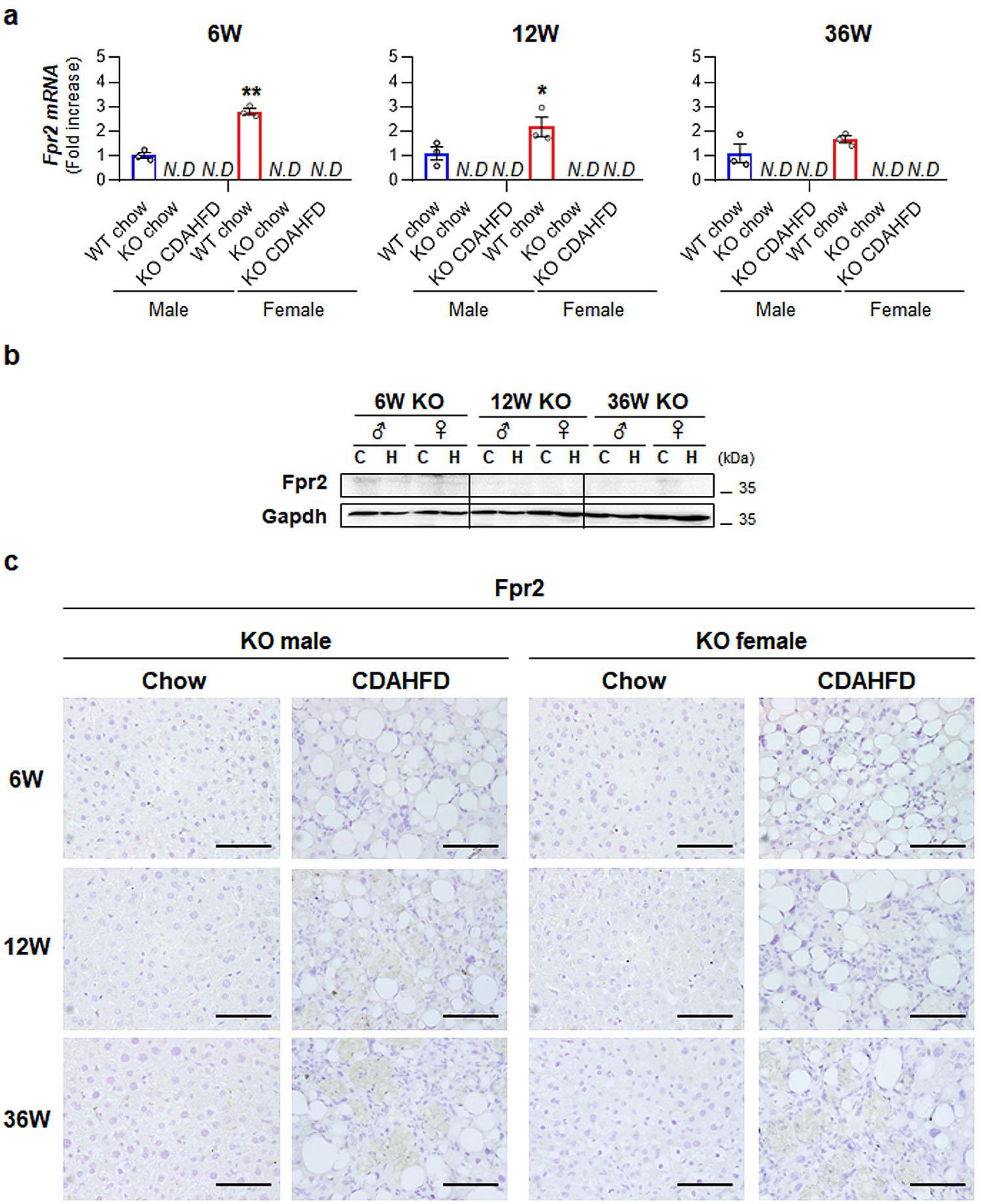

Supplementary Figure 6. Analysis of hepatic Fpr2 expression in WT and KO mice
(a) qRT-PCR analysis for *Fpr2* in livers from WT male and female mice treated with chow and KO male and female mice fed either chow or CDAHFD. Data represent the mean ± S.E.M (n ≥ 3 /group, *p<0.05, **p<0.005 vs Chow-WT male). (b) Western blot assays for Fpr2 in whole liver extracts from KO mice fed either chow or CDAHFD. Each lane contains protein lysates pooled from representative 3 mice per group with equal concentration. Gapdh was used as internal control. Data shown represent one of three experiments with similar results. (c) Representative Fpr2-immunostained liver images from the KO groups (Scale bar, 50μm). Gray circles represent individual data points. See Supplementary Data for statistical details.

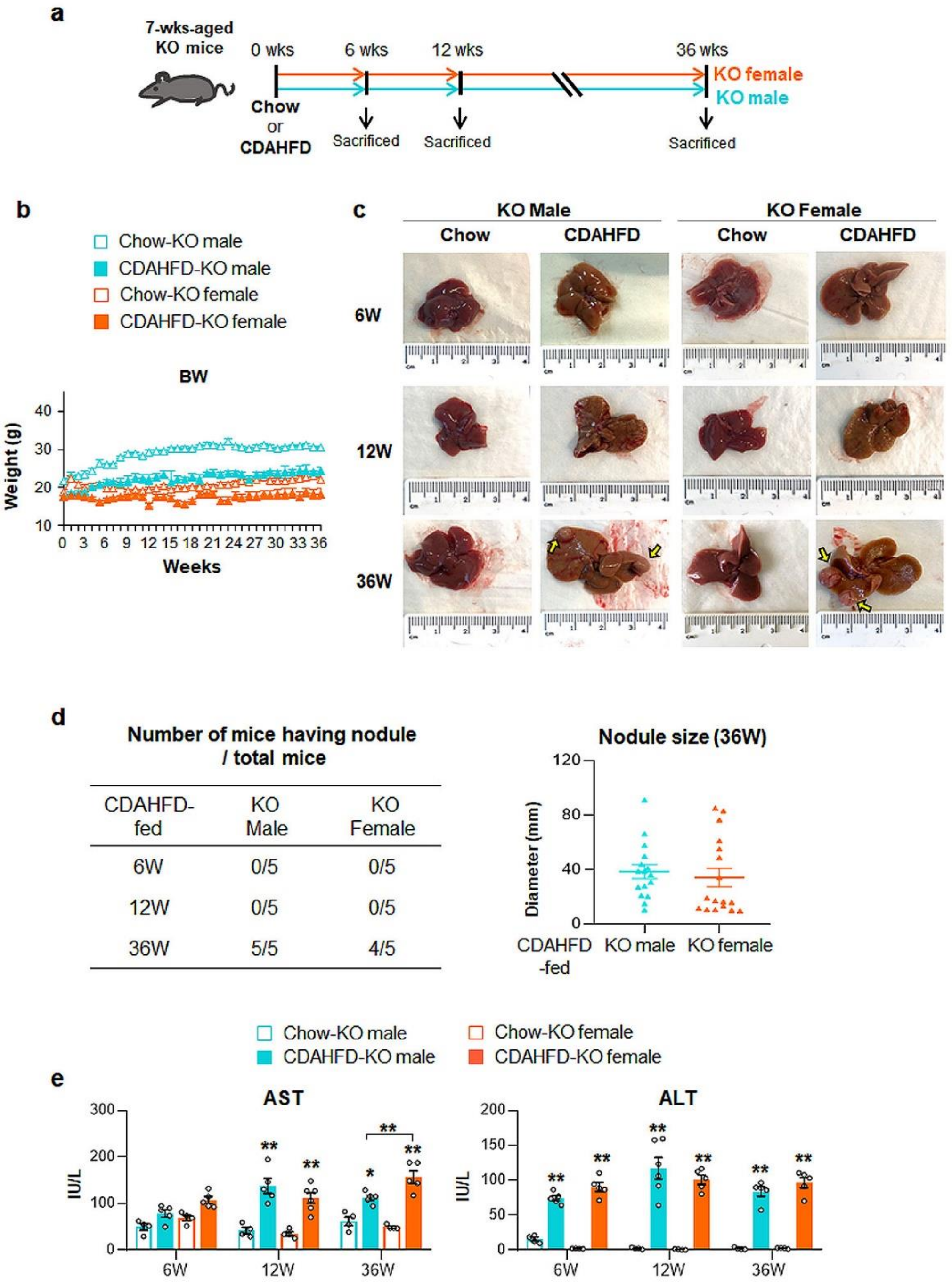

Supplementary Figure 7. Fpr2 Deficiency promotes severe hepatic damages in female mice exposed to CDAHFD

(a) A scheme for animal experiment in which 7-week-old KO male and female mice were fed either chow or CDAHFD for 6, 12 and 36 weeks. (b) The body weight changes of these mice for 36 weeks. Data represent the mean ± S.E.M. (c) Representative macroscopic appearance of livers from each group. Yellow arrows indicate nodules. (d) The number of KO mice having nodule was counted and diameter of nodules from these mice was measured and plotted. Data represent the mean ± S.E.M. (e) Levels of serum AST and ALT of these mice. Data represent the mean ±

S.E.M. ($n \geq 3$ /group, *$p<0.05$, **$p<0.005$ vs own control). Gray circles represent individual data points. See Supplementary Data for statistical details.

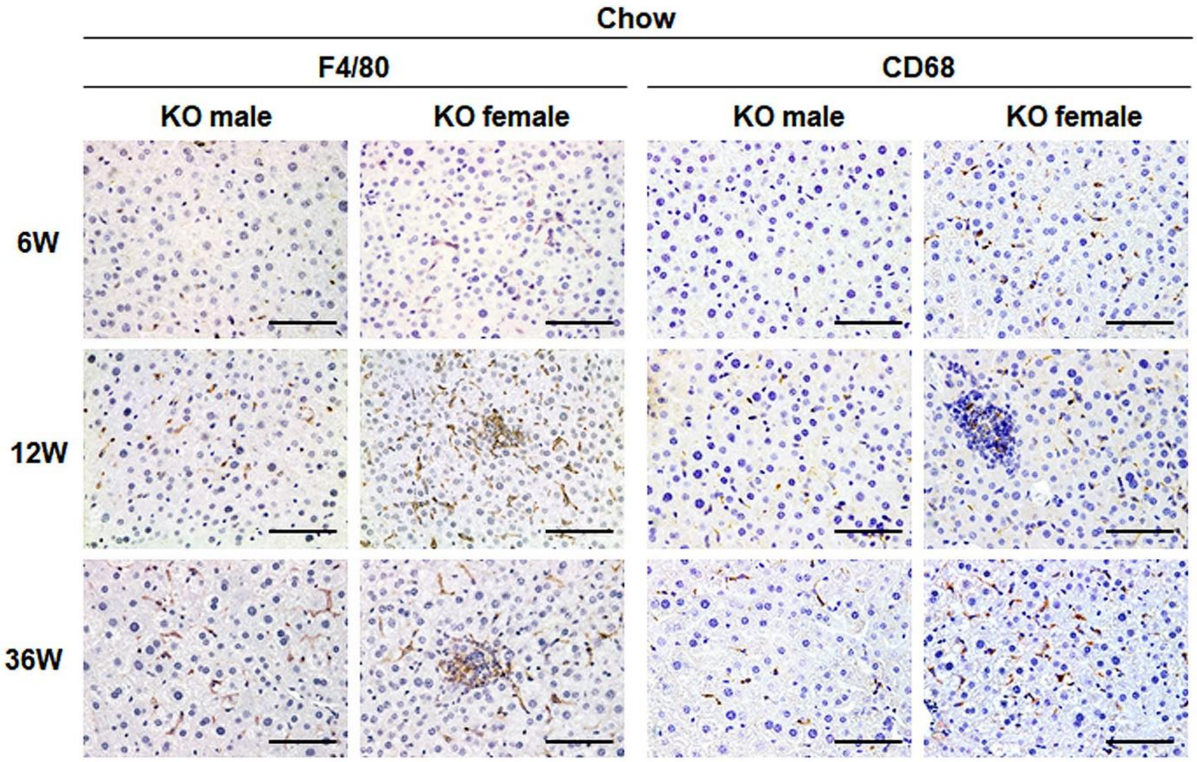

Supplementary Figure 8. Examination of inflammatory cells in the livers of KO mice treated with chow diet

Representative images of F4/80- (left panel, n=4) or CD68-stained (right panel, n=4) liver sections from the Chow-KO groups (Scale bar, 50μm).

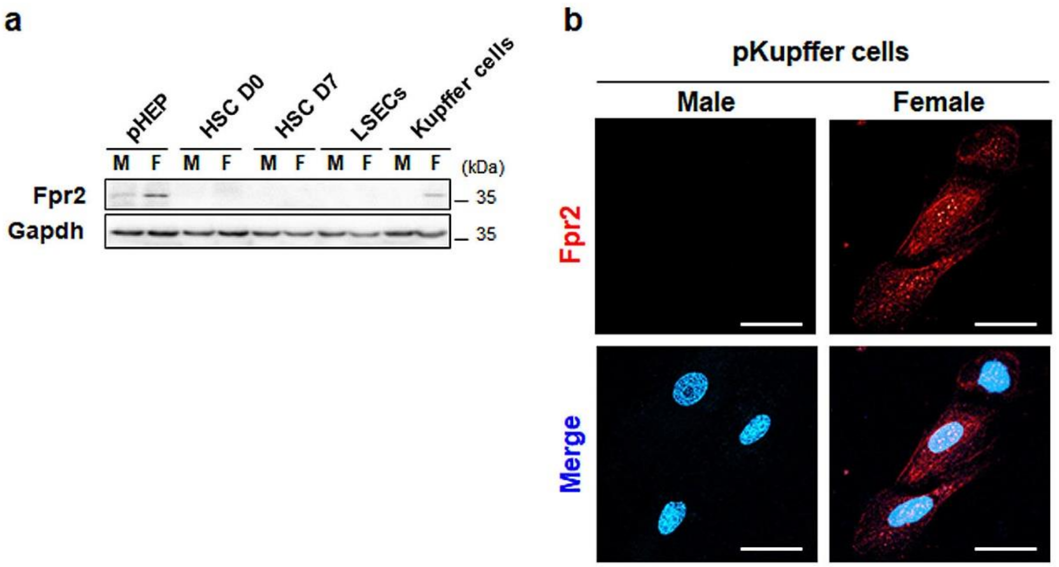

Supplementary Figure 9. Analysis of Fpr2 expression in liver cells

(a) Western blot analysis for Fpr2 in primary hepatocytes (pHEP), quiscient hepatic stellate cells (HSC D0) and culture-activated HSC (HSC D7), liver sinusoidal endothelial cells (LSEC) and Kupffer cells isolated from WT male and female mice (n≥ 2 /group). Gapdh was used as internal control. Data shown represent one of the three experiments with similar results. (b) Representative confocal images of Fpr2 (red) in primary Kupffer cells isolated from these mice. DAPI (blue) was used as nuclear counterstaining (Scale bar, 20μm). Data shown represent one of three experiments with similar results.

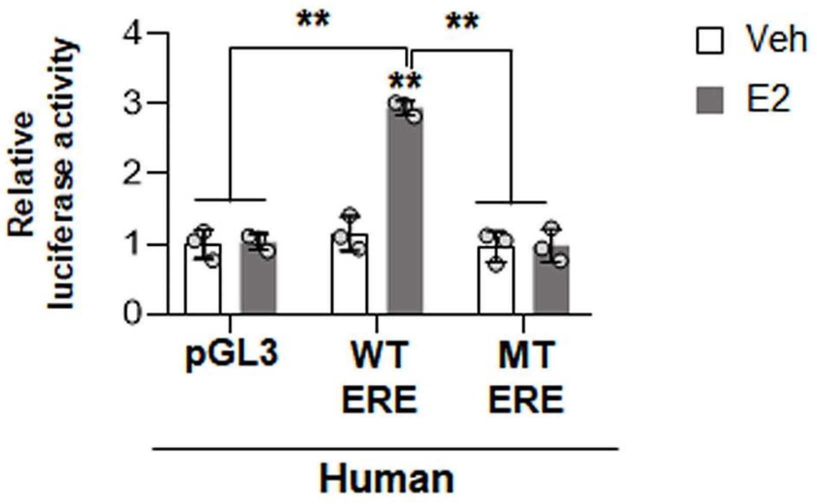

Supplementary Figure 10. Estradiol directly regulates expression of human FPR2

Dual-luciferase reporter assay was performed to verify biding interaction between estradiol and estrogen response element (ERE) in the promoter region of human FPR2 gene. HepG2 cells transfected with a pGL3 vector having either WT- or mutant (MT-) ERE on the promoter region of Fpr2. These cells were exposed to either 100 nM E2 or vehicle (Veh). Mean ± S.E.M. results are graphed (*p<0.05, **p<0.005 vs Veh). Gray circles represent individual data points. See Supplementary Data for statistical details.

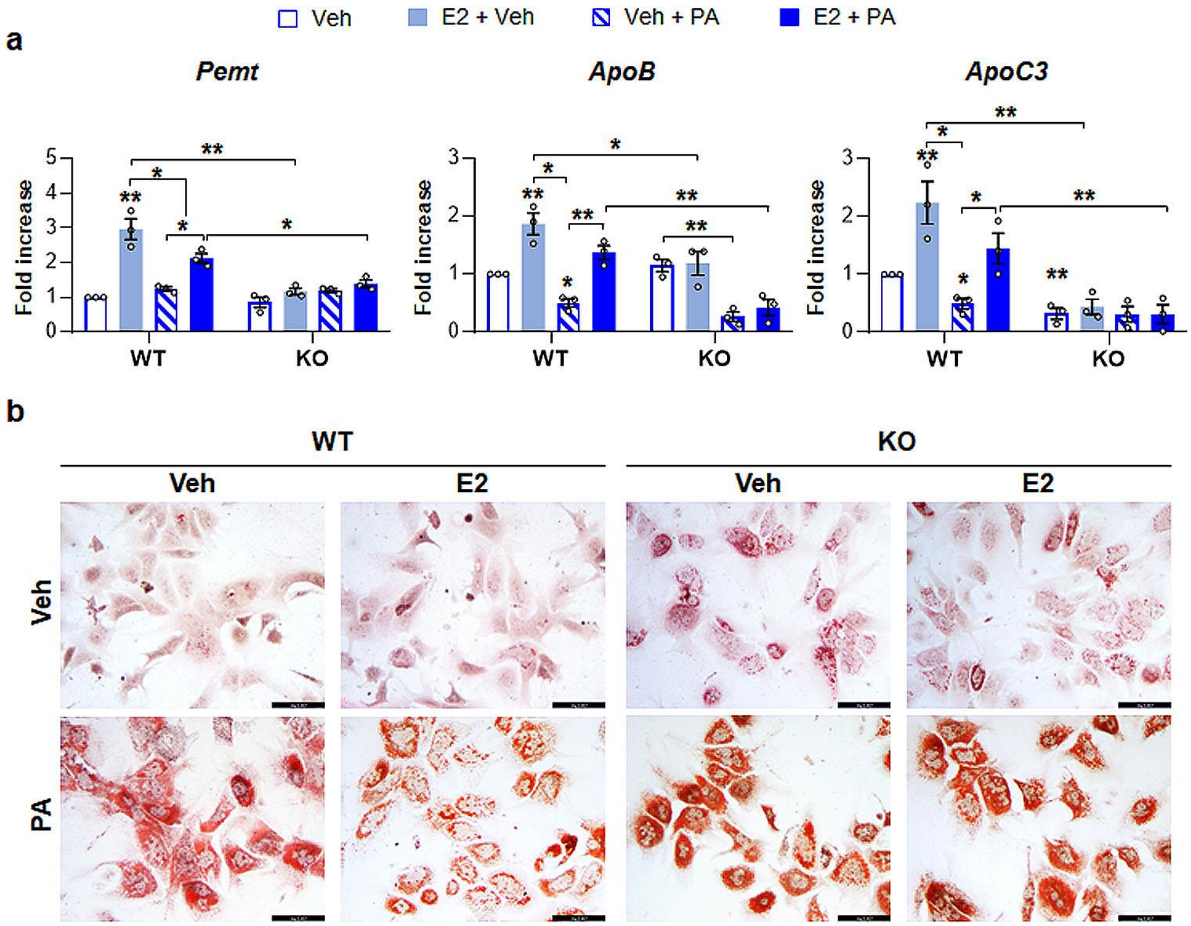

Supplementary Figure 11. Fpr2 enhances the expression of VLDL secretion-related genes by upregulating *Pemt*, and lowers fat accumulation in PA-treated hepatocytes

(a) qRT-PCR analysis for *Pemt, ApoB, and ApoC3* and in pHEPs from WT or KO male mice. These cells treated with either vehicle or 100 nM of E2 were exposed to 250 μM of PA for 24hours. The mean ± S.E.M. results obtained from three repetitive experiments are graphed (**p*<0.05, ***p*<0.005 vs WT-Veh). (b) Oil red O staining for lipid droplet in these cells. Representative images are shown (Scale bars, 20μm). Data shown represent one of three experiments with similar results. Gray circles represent individual data points. See Supplementary Data for statistical details.

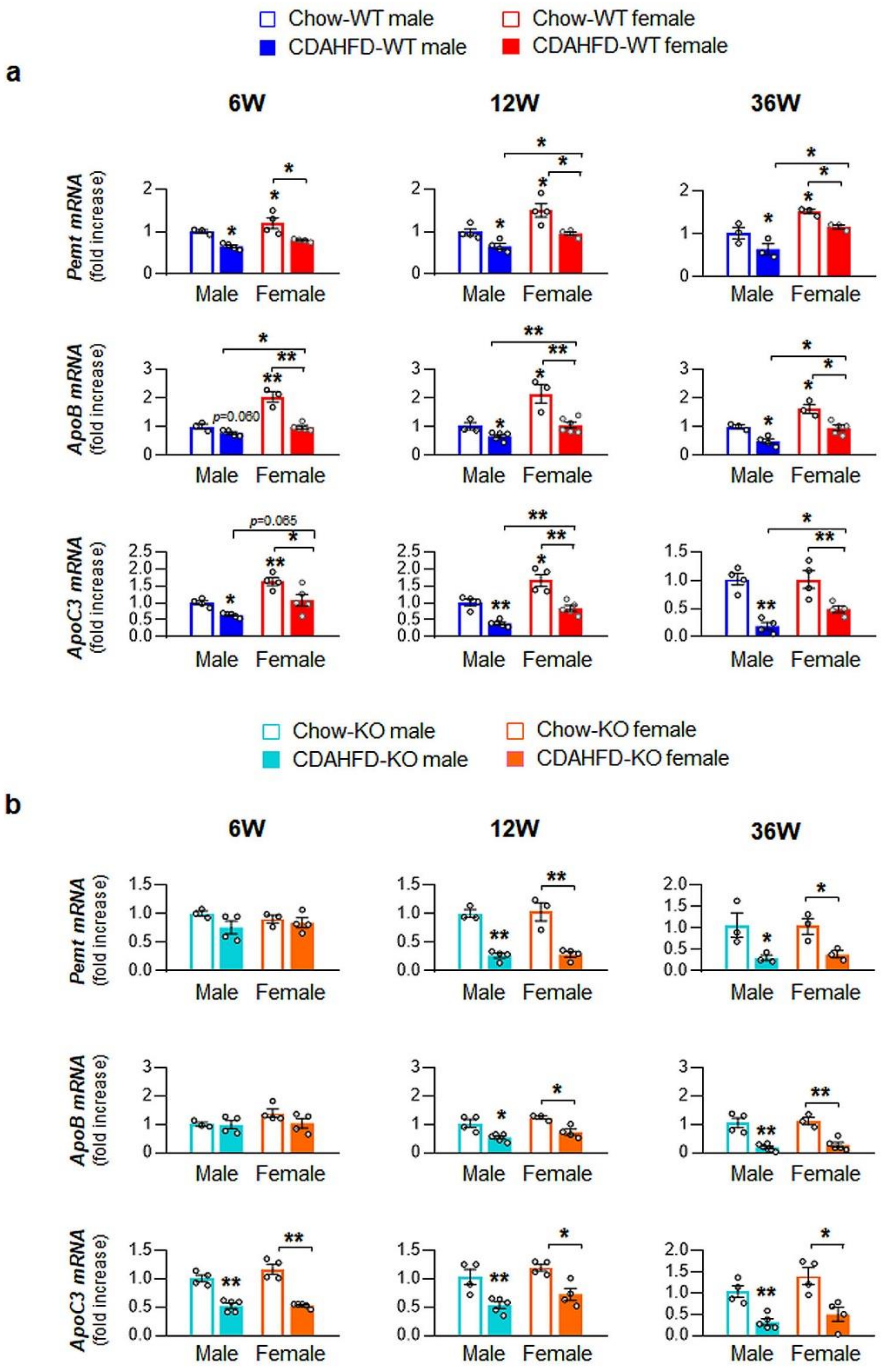

Supplementary Figure 12. Analysis of the RNA expressions of *Pemt* and VLDL-secretion-related markers in WT and KO mice

(a) qRT-PCR analysis for *Pemt, ApoB, and ApoC3* from WT or (b) KO male and female mice treated with chow or CDAHFD for 6, 12 and 36 weeks. Data represent the mean ± S.E.M. (n ≥ 3 /group, *p<0.05, **p<0.005 vs own control). Gray circles represent individual data points. See Supplementary Data for statistical details.

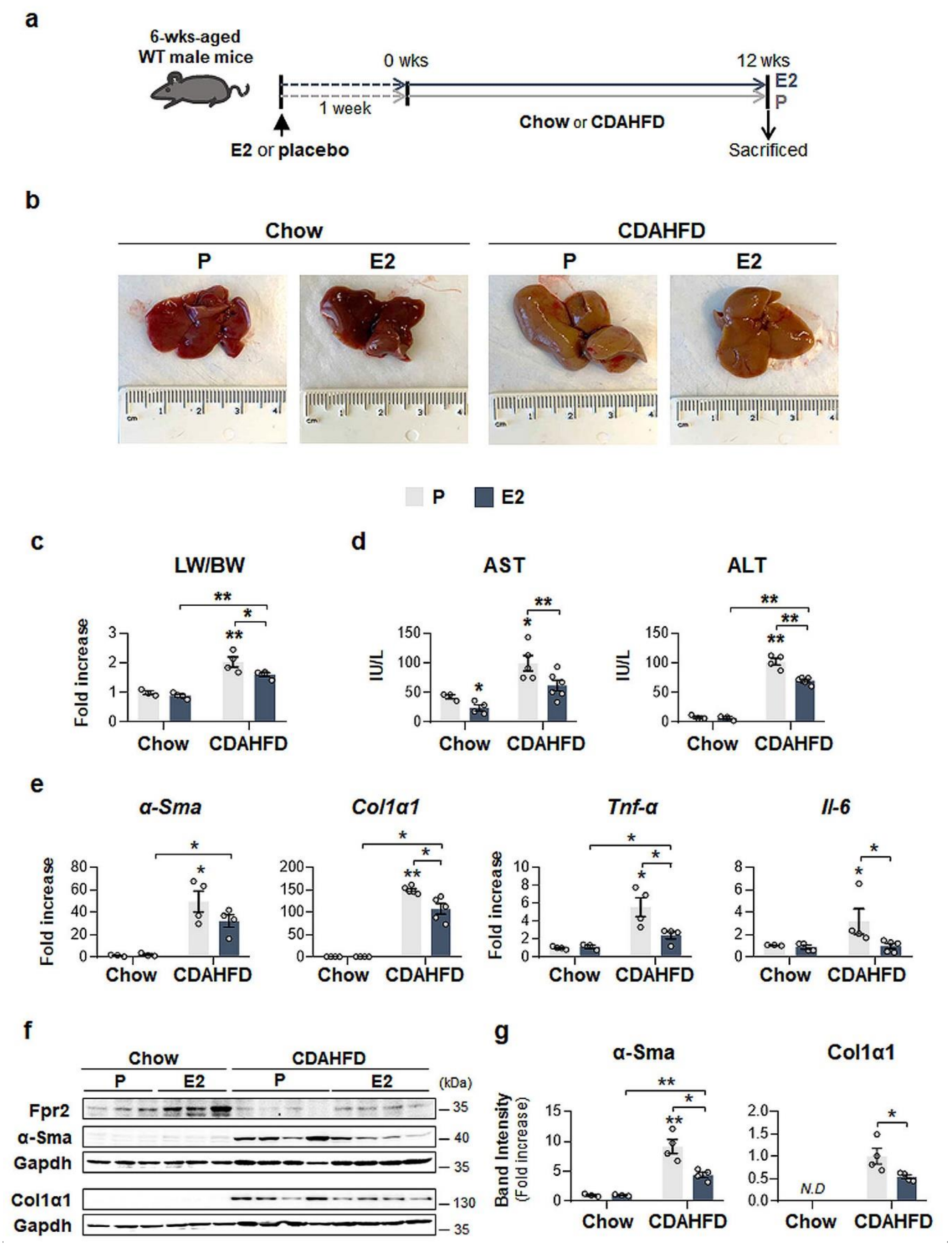

Supplementary Figure 13. Estradiol attenuates CDAHFD-induced damage in male mice

(a) A scheme for animal experiments in which 6-week-old WT male mice were supplemented with placebo (P) or estradiol (E2) pellet, and then fed either chow or CDAHFD for 12 weeks. (b) Representative macroscopic appearance of livers from these mice. (c) LW/BW ratios and (d)

serum AST and ALT level in these mice. (e) qRT-PCR analysis for hepatic *α-Sma*, *Col1α1*, *Tnf-α* and *Il-6* in representative mice per each group. (f) Western blot analysis of hepatic Fpr2, α-Sma, and Col1α1 expression representative mice per each group. Gapdh was used as internal control. Data shown represent one of the three experiments with similar results. (g) Cumulative densitometric analysis for α-Sma and Col1α1 in the livers of representative mice per each group. Band densities were normalized to the expression level of Gapdh. All data represent the mean ± S.E.M. ($n \geq 3$ /group, *$p<0.05$, **$p<0.005$ vs Chow-P). Gray circles represent individual data points. See Supplementary Data for statistical details.

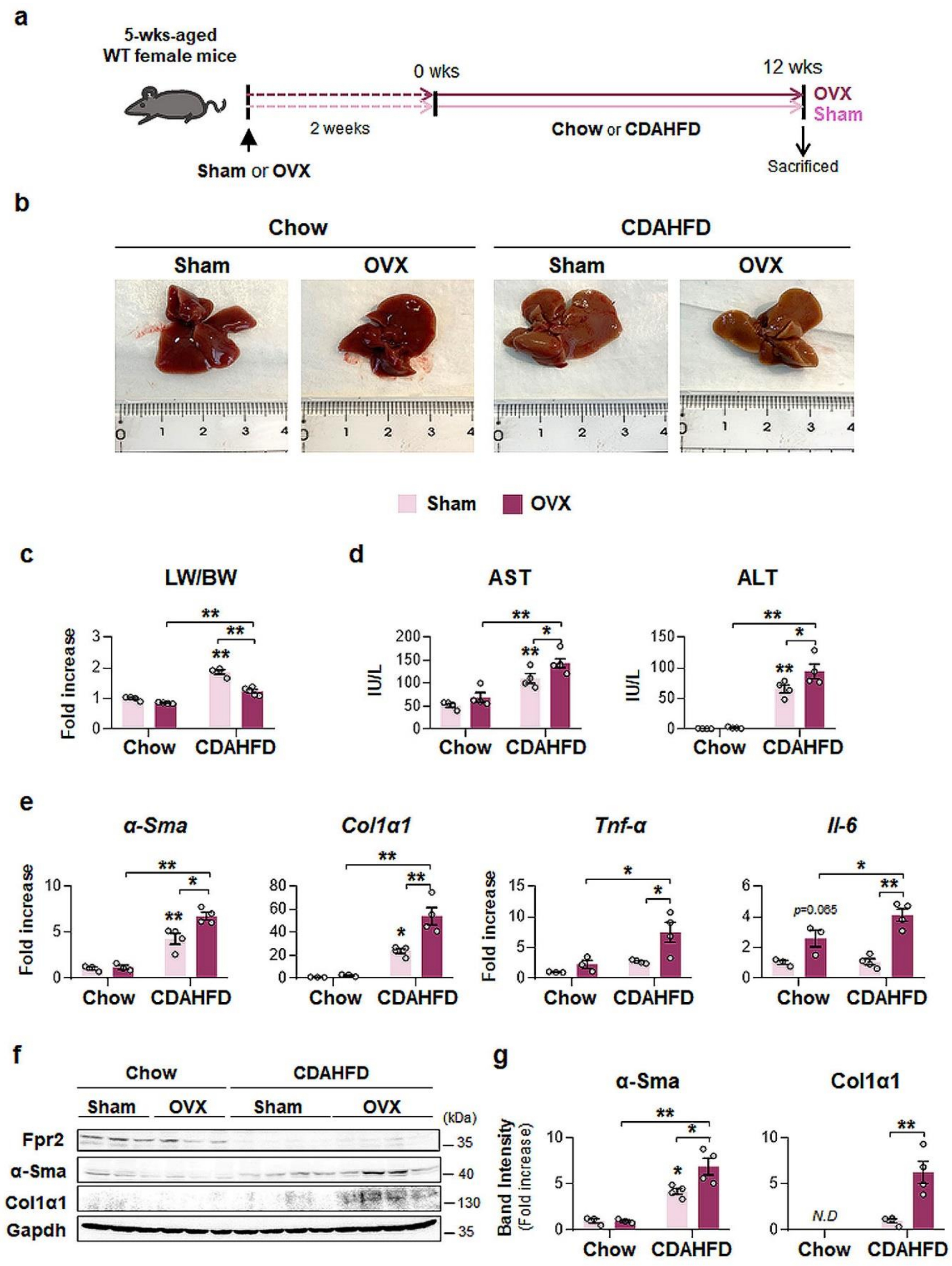

Supplementary Figure 14. Ovariectomy accelerates NASH progression in CDAHFD-fed female mice

(a) A scheme for animal experiments in which 5-week-old WT female mice underwent sham-operated (Sham) or ovariectomy (OVX), and then were fed either chow or CDAHFD for 12 weeks. (b) Representative macroscopic appearance of livers from these mice. (c) LW/BW ratios and (d) levels of serum AST and ALT in these mice. (e) qRT-PCR analysis for hepatic *α-Sma*, *Col1α1*, *Tnf-α* and *Il-6* in representative mice per each group. (f) Western blot analysis of hepatic Fpr2, α-Sma, and Col1α1 expression representative mice per each group. Gapdh was used as internal control. Data shown represent one of the three experiments with similar results. (g) Cumulative densitometric analysis for α-Sma and Col1α1 in the livers of representative mice per each group. Band densities were normalized to the expression level of Gapdh. All data represent the mean ± S.E.M. ($n \geq 3$ /group, *$p<0.05$, **$p<0.005$ vs Chow-Sham). Gray circles represent individual data points. See Supplementary Data for statistical details.

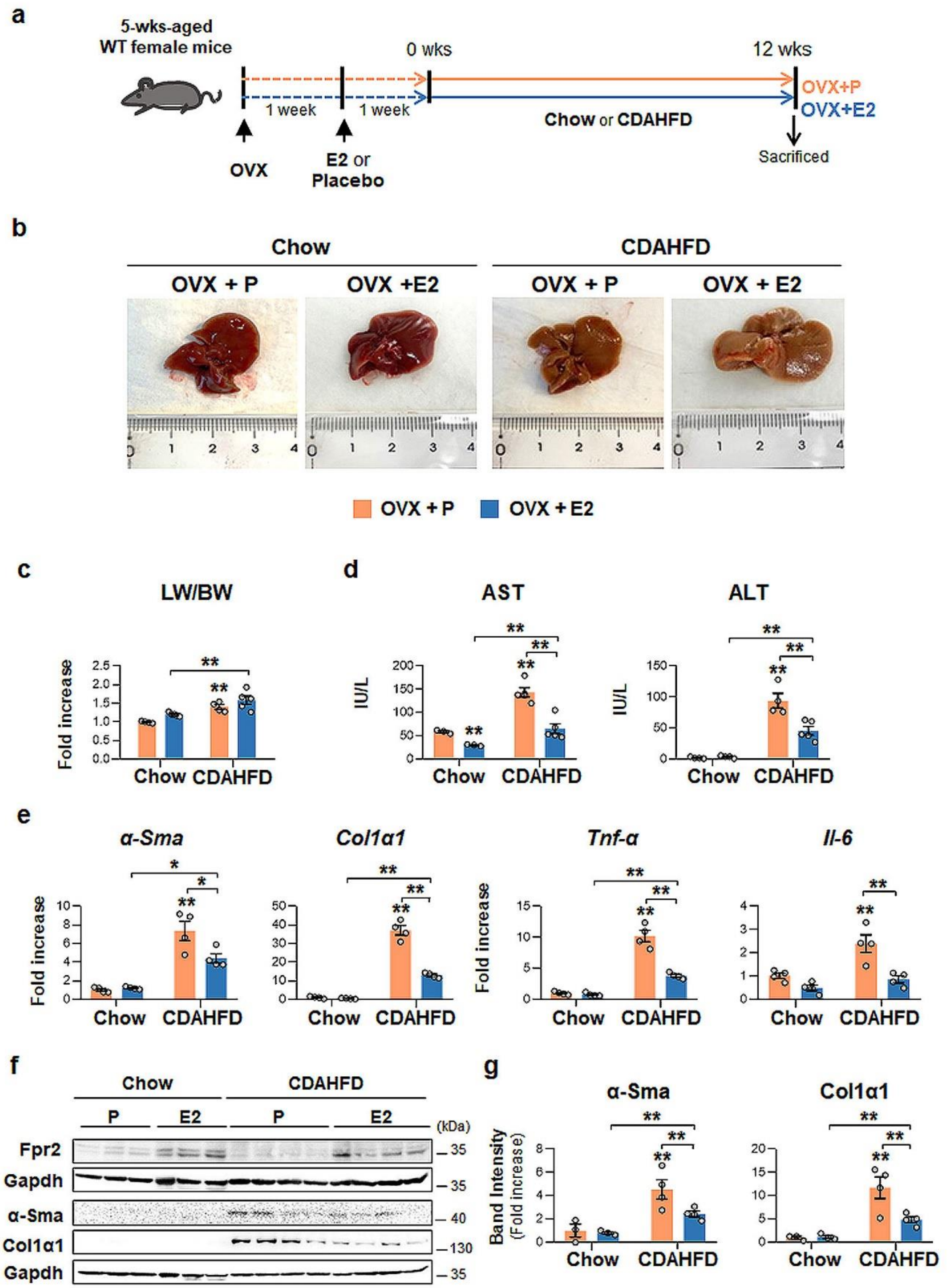

Supplementary Figure 15. Estradiol treatment alleviates CDAHFD-induced damage in ovariectomized female mice

(a) A scheme for animal experiments in which 5-week-old ovariectomized WT female mice were supplemented with placebo (OVX-P) or estradiol (OVX-E2) pellet, and then fed either chow or CDAHFD for 12 weeks. (b) Representative macroscopic appearance of livers from these mice. (c) LW/BW ratios and (d) levels of serum AST and ALT in these mice. (e) qRT-PCR analysis for hepatic *α-Sma*, *Col1α1*, *Tnf-α* and *Il-6* in livers from these mice. (f) Western blot analysis of Fpr2, α-Sma, and Col1α1 in three representative mice per each group. Gapdh was used as internal control. Data shown represent one of the three experiments with similar results. (g) Cumulative densitometric analysis for α-Sma and Col1α1 are displayed. Band densities were normalized to the expression level of Gapdh. All data represent the mean ± S.E.M. (n ≥ 3 /group, *p<0.05, **p<0.005 vs Chow-OVX-P). Gray circles represent individual data points. See Supplementary Data for statistical details.

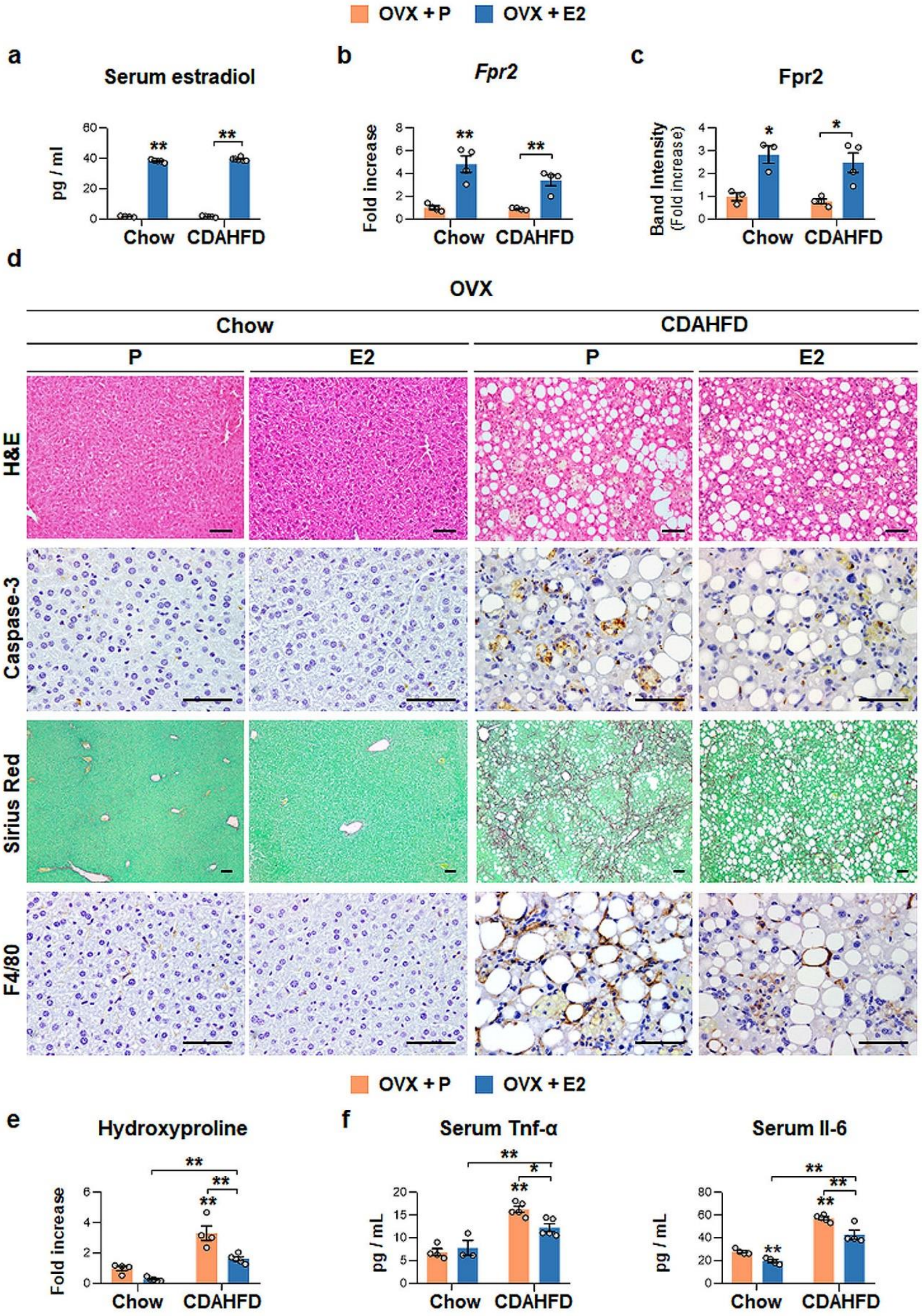

Supplementary Figure 16. Estradiol implantation improves CDAHFD-induced damage in female mice with OVX by upregulating Fpr2

(a) Amount of serum estradiol in, (b) qRT-PCR and (c) cumulative densitometric analysis for hepatic Fpr2 in placebo (OVX-P) or estradiol (OVX-E2)-supplemented female mice fed either chow or CDAHFD for 12 weeks. Band densities were normalized to the expression level of Gapdh, an internal control. Data represent the mean ± S.E.M. (n ≥ 3 /group, *p<0.05, **p<0.005 vs Chow-P). (d) Representative images of H&E-, Caspase-3-, Sirius red-, and F4/80-stained liver sections from these mice (Scale bar: 50μm). (e) Hepatic hydroxyproline content and (f) serum Tnf-α and Il-6 level in these mice. Data represent the mean ± S.E.M. (n ≥ 3 /group, *p<0.05, **p<0.005 vs Chow-P). Gray circles represent individual data points. See Supplementary Data for statistical details.

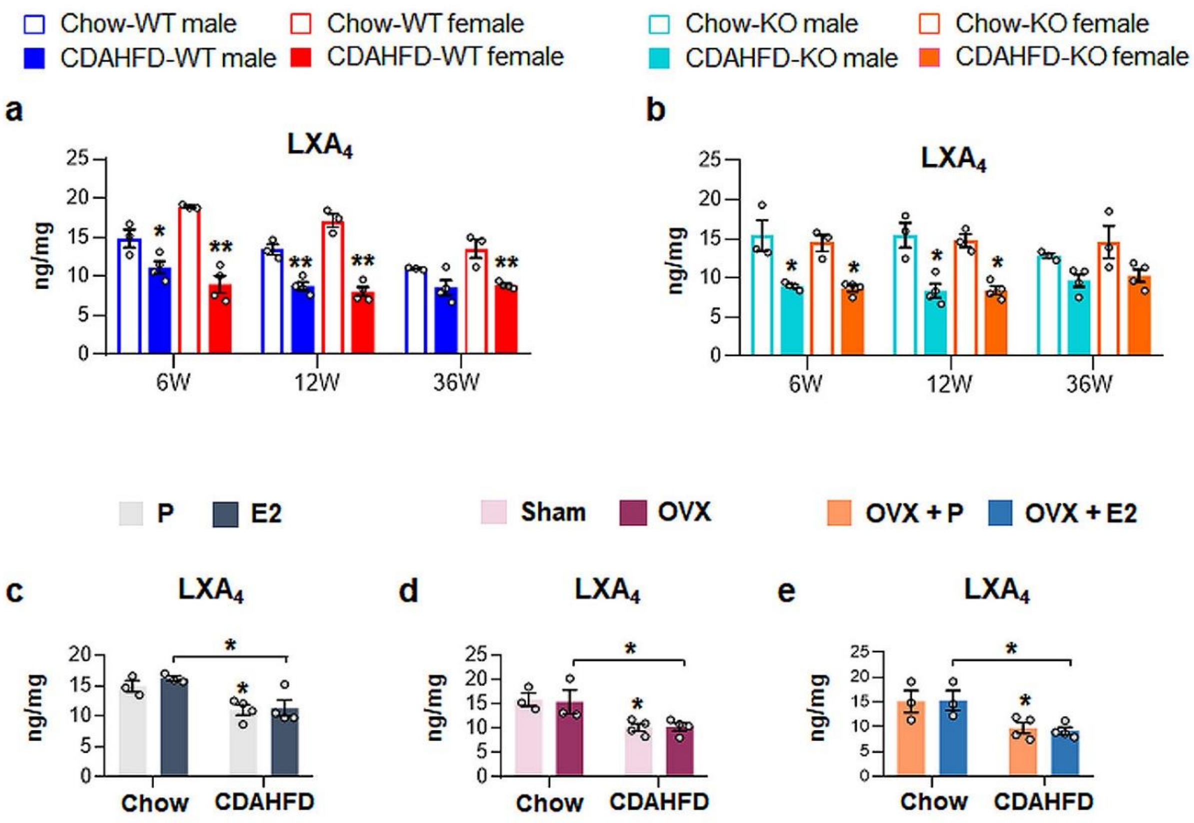

Supplementary Figure 17. Analysis of hepatic LXA_4 in experimental mouse model

(a) Amount of hepatic LXA_4 in WT male and female mice and (b) in KO male and female mice fed either chow or CDAHFD for 6, 12 and 36 weeks. Data represent the mean ± S.E.M. (n ≥ 3 /group, *p<0.05, **p<0.005 vs own control). (c) Levels of hepatic LXA_4 in WT male mice supplemented with placebo (P) or estradiol (E2) pellet, and (d) in WT female mice underwent sham-operated (Sham) or ovariectomy (OVX) and (e) in ovariectomized-WT female mice supplemented with placebo (OVX-P) or estradiol (OVX-E2). Mice in (c)-(e) fed either chow or CDAHFD for 12 weeks, and data in (c)-(e) represent the mean ± S.E.M. (n ≥ 3 /group, *p<0.05 vs Chow groups). Gray circles represent individual data points. See Supplementary Data for statistical details.

Supplementary Table

List of mouse primer sequences used for QRT-PCR

Fpr2	5’-CACAGGAACCGAAGAGTGTAAG-3’	5’-CACCATTGAGAGGATCCACAG-3’
Tnf-α	5’- TCGTAGCAAACCACCAAGTG-3’	5’- ATATAGCAAATCGGCTGACG-3’
Il-6	5’-AAGCCAGAGTCCTTCAgAGAGATACAG3’	5’-ATGAATTGGATGGTCTTGGTCCTTAG -3’
α-sma	5’-AAACAGGAATACGACGAAG-3’	5’-CAGGAATGATTTGGAAAGGA-3’
Col1α1	5’-GAGCGGAGAGTACTGGATCG-3’	5’-GCTTCTTTTCCTTGGGGTTC-3’
Erα	5’-AGGGAAGCTCCTATTTGCTCC-3’	5’-CGGTGGATGTGGTCCTTCTCT-3’
G6pc	5’-TCCTCCTCAGCCTATGTCTGCATTC-3’	5’-GAGAGAAGAATCCTGGGTCTCCTTG-3’
Pemt	5’-CGAGATGGGAGCAGAGAACT-3’	5’-ATCTTGGGCTGGCTCATCAT-3’
ApoB	5’-TCTACAACTGGTCAGCCTCCTACAC-3’	5’-TTGTGTAGAGATCCATCACAGGAC-3’
ApoC3	5’-CGCTAAGTAGCGTGCAGGA-3’	5’-GGGATCTGAAGTGATTGTCCA-3’
9s	5’-GGGCCTGAAGATTGAGGATT-3’	5’-CGGGCATGGTGAATAGATTT-3’
