# Supplementary material for: Formyl peptide receptor 2 determines sex-specific differences in the progression of nonalcoholic fatty liver disease and steatohepatitis
Source: Nat Commun. 2022 Jan 31;13:578. doi: 10.1038/s41467-022-28138-6 (PMC8803937; doi:10.1038/s41467-022-28138-6)
Supplement: Supplementary file 3 — Reporting Summary [file 41467_2022_28138_MOESM3_ESM.pdf]

nature research

Corresponding author(s): Youngmi Jung

Last updated by author(s): Dec 7, 2021

Reporting Summary

Nature Research wishes to improve the reproducibility of the work that we publish. This form provides structure for consistency and transparency in reporting. For further information on Nature Research policies, see our Editorial Policies and the Editorial Policy Checklist.

Statistics

For all statistical analyses, confirm that the following items are present in the figure legend, table legend, main text, or Methods section.

n/a	Confirmed	
☐	☒	The exact sample size (n) for each experimental group/condition, given as a discrete number and unit of measurement
☐	☒	A statement on whether measurements were taken from distinct samples or whether the same sample was measured repeatedly
☐	☒	The statistical test(s) used AND whether they are one- or two-sided<br>*Only common tests should be described solely by name; describe more complex techniques in the Methods section.*
☒	☐	A description of all covariates tested
☒	☐	A description of any assumptions or corrections, such as tests of normality and adjustment for multiple comparisons
☐	☒	A full description of the statistical parameters including central tendency (e.g. means) or other basic estimates (e.g. regression coefficient) AND variation (e.g. standard deviation) or associated estimates of uncertainty (e.g. confidence intervals)
☐	☒	For null hypothesis testing, the test statistic (e.g. F, t, r) with confidence intervals, effect sizes, degrees of freedom and P value noted<br>*Give P values as exact values whenever suitable.*
☒	☐	For Bayesian analysis, information on the choice of priors and Markov chain Monte Carlo settings
☒	☐	For hierarchical and complex designs, identification of the appropriate level for tests and full reporting of outcomes
☐	☒	Estimates of effect sizes (e.g. Cohen's d, Pearson's r), indicating how they were calculated

Our web collection on statistics for biologists contains articles on many of the points above.

Software and code

Policy information about availability of computer code

Data collection	CS analyzer 4.0 program (Version1.0.3, ATTO) used to collect western blot data. ZEN 2.3 (Carl Zeiss Inc.,blue edition) was used to display IF images, Cell sense Entry (version 1.14, olympus) was used to display of stained images.
Data analysis	CS analyzer 4.0 program (Version1.0.3, ATTO) was used to analyze band density of western blotting, TRNASFAC database (version 8.0) was used to predict ERE sequence of FPR2 promoter, IBM SPSS 21.0 and GraphPad Prism 8 (GraphPad Software Inc.) was used for statistical analysis and used for generation of all graphs.

For manuscripts utilizing custom algorithms or software that are central to the research but not yet described in published literature, software must be made available to editors and reviewers. We strongly encourage code deposition in a community repository (e.g. GitHub). See the Nature Research guidelines for submitting code & software for further information.

Data

Policy information about availability of data

All manuscripts must include a data availability statement. This statement should provide the following information, where applicable:
- Accession codes, unique identifiers, or web links for publicly available datasets
- A list of figures that have associated raw data
- A description of any restrictions on data availability

Source data are provided with this manuscript. The datasets used in this study are available in the GEO database under accession code GSE66676 [https://www.ncbi.nlm.nih.gov/geo/query/acc.cgi?acc=GSE66676].

Field-specific reporting

Please select the one below that is the best fit for your research. If you are not sure, read the appropriate sections before making your selection.

- [x] Life sciences
- [ ] Behavioural & social sciences
- [ ] Ecological, evolutionary & environmental sciences

For a reference copy of the document with all sections, see nature.com/documents/nr-reporting-summary-flat.pdf

Life sciences study design

All studies must disclose on these points even when the disclosure is negative.

Sample size	At least three independent experiments were repeated for in vitro experiment, . For animal study, group sizes were selected empirically based upon prior knowledge of the intra-group variation of NAFLD animal models in order to ensure adequate statistical power (n ≥3). Exact information on the sample numbers being analyzed can be found in Supplementary Data.
Data exclusions	None
Replication	At least three times experiments have been conducted independently and obtained similar results.
Randomization	For all animal experiments, mice were randomly assigned to different experimental groups.
Blinding	After setting up the in vitro experiments, code number was given to each sample and blinding test has been conducted on these samples. Blinding method was also used for animal studies. The investigators were blinded during data collection and analysis.

Reporting for specific materials, systems and methods

We require information from authors about some types of materials, experimental systems and methods used in many studies. Here, indicate whether each material, system or method listed is relevant to your study. If you are not sure if a list item applies to your research, read the appropriate section before selecting a response.

Materials & experimental systems

n/a	Involved in the study	
☐	☒	Antibodies
☐	☒	Eukaryotic cell lines
☒	☐	Palaeontology and archaeology
☐	☒	Animals and other organisms
☒	☐	Human research participants
☒	☐	Clinical data
☒	☐	Dual use research of concern

Methods

n/a	Involved in the study	
☒	☐	ChIP-seq
☒	☐	Flow cytometry
☒	☐	MRI-based neuroimaging

Antibodies

Antibodies used	Antibodies were used for western blot analysis and immunostaining. <br>For western blot analysis, we used: Fpr2 (diluted 1:1000; NLS1878; Novus Biologicals), α-Sma (diluted 1:1000; A5228; Sigma-Aldrich), Col1α1 (diluted 1:1000; NBP1-30054; Novus Biologicals, LLC, USA), cleaved caspase-3 (diluted 1:1000; 9661; Cell signaling technology, Danvers, MA, USA), caspase-3 (diluted 1:1000; 9662; Cell Signaling), glyceraldehyde 3-phosphate dehydrogenase antibody (Gapdh; diluted 1:1,000; MCA4739; AbD Serotec, Oxford, UK), HRP-conjugated anti-rabbit IgG (diluted 1:5000; ADI-SAB-300-J; Enzo Life Sciences, Inc., Farmingdale, NY, USA), HRP-conjugated anti-mouse IgG (diluted 1:5000; ADI-SAB-100-J; Enzo Life Sciences, Inc., Farmingdale, NY, USA) <br>For immunostaining, we used: active caspase-3 (diluted 1:500; AF835; R&D systems, Minneapolis, MN, USA), F4/80 (diluted 1:500; ab6640; Abcam, Cambridge, MA, USA), Fpr2 (diluted 1:200; ab203129; Abcam), albumin (diluted 1:500; sc-69873; Santacruz), Polymer horseradish peroxidase (HRP) anti-rabbit (diluted 1:500; K4003; Dako), HRP anti-rat IgG (diluted 1:200; A110-105P; BETHYL, Montgomery, Texas, USA), Alexa Fluor 568 goat anti-rabbit IgG (diluted 1:100; A-11011; Invitrogen), Alexa Fluor 488 chicken anti-mouse IgG (diluted 1:100; A-21200; Invitrogen).
Validation	All antibodies were sold by the manufacturer with validation data and citations, and they detected the specified targets in our study as expected. We based specificity on their provided description and data sheets. Specificity was confirmed by obtaining the expected pattern of tissue staining with the respective antibodies. <br>Fpr2, applications: WB, EM, Flow, ICC/IF, IHC; species reactivity: Human, Mouse, Rat, Bacteria

https://www.novusbio.com/products/fprl1-fpr2-antibody_nls1878

α-Sma, applications: IHC, ELISA, IF, microarray, WB; species reactivity: Human, Frog, Sheep, Chicken, Goat, Bovine, Rat, Guinea pig, Mouse, Canine, Rabbit, Snake
https://www.sigmaaldrich.com/KR/ko/product/sigma/a5228

Col1α1, applications: WB. ICC/IF, IHC; species reactivity: Human, Mouse, Rat, Amphibian, Avian, Mammal, Sheep
https://www.novusbio.com/products/collagen-i-alpha-1-antibody_nbp1-30054

Cleaved caspase-3, applications: WB, IP, IHC, IF, Flow; species reactivity: Human, Mouse, Rat, Monkey
https://www.cellsignal.com/products/primary-antibodies/cleaved-caspase-3-asp175-antibody/9661

Caspase-3, applications: WB, IP, IHC; species reactivity: Human, Mouse, Rat, Monkey
https://www.cellsignal.com/products/primary-antibodies/caspase-3-antibody/9662

Glyceraldehyde 3-phosphate dehydrogenase antibody, applications: ELISA, IF, IHC, IP, WB; species reactivity: Human, Pig, Dog, Cat, Rat, Mouse, Xenopus, Tube-nosed Bat, Chicken, Sheep, African green monkey, Crucian crap
https://www.bio-rad-antibodies.com/monoclonal/rabbit-lapine-gapdh-antibody-6c5-mca4739.html?f=purified

HRP-conjugated anti-rabbit IgG, applications: ELISA, IHC, WB; species reactivity: Rabbit
https://www.enzolifesciences.com/ADI-SAB-300/goat-anti-rabbit-igg-polyclonal-antibody-hrp-conjugate/

HRP-conjugated anti-mouse IgG, applications: ELISA, IHC, WB; species reactivity: Mouse
https://www.enzolifesciences.com/ADI-SAB-100/goat-anti-mouse-igg-f-ab-2-polyclonal-antibody-hrp-conjugate/

Active caspase-3, applications: IHC; species reactivity: Human, Mouse
https://www.rndsystems.com/products/human-mouse-active-caspase-3-antibody_af835

F4/80, applications: Flow, ICC/IF; species reactivity: Mouse
https://www.abcam.com/f480-antibody-cia3-1-macrophage-marker-ab6640.html

Fpr2, applications: IHC, Flow; species reactivity: Mouse, Rat, Human
https://www.abcam.com/fprl1rfp-antibody-ab203129.html

Albumin, applications: WB, IP, IF; species reactivity: Human
https://www.scbt.com/ko/p/alb-antibody-sab117

Polymer horseradish peroxidase (HRP) anti-rabbit, applications: IHC; species reactivity: Rabbit
https://www.agilent.com/en/product/immunohistochemistry/visualization-systems/envision-systems/envision-single-reagent-(hrp-rabbit)-76782

HRP anti-rat IgG, applications: ELISA, ICC, IHC, WB; species reactivity: Rat
https://www.fortislife.com/products/secondary-antibodies/goat-anti-rat-igg-heavy-and-light-chain-antibody-hrp-conjugated/A110-105P

Alexa Fluor 568 goat anti-rabbit IgG, applications:WB, IHC, ICC/IF, Flow ; species reactivity: Rabbit
https://www.thermofisher.com/antibody/product/Goat-anti-Rabbit-IgG-H-L-Cross-Adsorbed-Secondary-Antibody-Polyclonal/A-11011

Alexa Fluor 488 chicken anti-mouse IgG, applications:WB, IHC, ICC/IF, Flow ; species reactivity: Mouse
https://www.thermofisher.com/antibody/product/Chicken-anti-Mouse-IgG-H-L-Cross-Adsorbed-Secondary-Antibody-Polyclonal/A-21200

Eukaryotic cell lines

Policy information about cell lines

Cell line source(s)	AML12 (mouse hepatocyte cell lines) were originally obtained from ATCC (CRL-2254). HepG2 cell, derived from the human hepatocellular carcinoma (HCC), were friendly presented from Dr. Sang-Woo Kim in Pusan National University(obtained from Korean Cell Line Bank, Seoul, Korea).
Authentication	The cell lines used in this paper from ATCC were not specifically re-authenticated.
Mycoplasma contamination	Cell lines were not tested for mycoplasma contamination but no indication of contamination was observed.
Commonly misidentified lines (See ICLAC register)	No commonly misidentified cell lines were used.

Animals and other organisms

Policy information about studies involving animals; ARRIVE guidelines recommended for reporting animal research

Laboratory animals	All mice housed with 12-hour light/dark cycle and allowed free access to normal food and water at an average temperature of 22°C ± 1°C and humidity of 50% ± 10%.<br><br>C57BL/6 mice were purchased from the Hyochang (Dae-gu, Korea). We obtain FPR2 Knockout mice (background 129S/SvEv x C57BL/6 strain) from Dr. Kim (Pusan National University School of Medicine, Yangsan, Korea). Both male and female mice were used for the study.<br><br>To mimic human-like NAFLD, 7-week-old male and female WT and KO mice fed normal chow-diet (Chow; M-diet; Optipharm.CO.,LTD, Cheongju, Korea) or choline-deficient, L-amino acid-defined high fat diet (CDAHFD; A06071302; Research diet, New Brunswick, NJ, USA) consisting 60 kcal% and 0.1% methionine for 6, 12, and 36 weeks, respectively.<br><br>To assess the protective effects of estrogen-mediated Fpr2 in vivo, 6-week-old WT male mice received either placebo (P) (n=10) or E2 pellets (E2; 0.36 mg; Innovative Research of America, Sarasota, FL, USA) (n=10) in the mid-ventral subcutaneous region. These male mice were divided into randomly four experimental groups and fed Chow or CDAHFD for 12 weeks<br><br>To confirm whether Fpr2 is involved in protective function of estrogen in female mice, 5-week-old C57BL/6 female mice underwent sham-surgery or ovariectomy as a surgical menopause model. These female mice were divided into randomly four experimental groups and fed Chow or CDAHFD for 12 weeks.<br><br>To double check the action of estradiol-mediated Fpr2 in the liver, female mice were treated with estradiol (OVX-E2) (n=10) or placebo pellet (OVX-P) (n=10) two weeks after they (5-week-old) underwent ovariectomy. Post one week after supplementation, these female mice were divided into randomly four experimental groups and fed Chow or CDAHFD for 12 weeks: Chow-OVX-P (n=4), CDAHFD-OVX-P (n=6), Chow-OVX-E2 (n=4), and CDAHFD-OVX-E2 (n=6). At the end of each time point, mice were sacrificed to collect blood and liver samples.
Wild animals	This study did not involve wild animals.
Field-collected samples	The study did not involve samples collected from the field.
Ethics oversight	All mouse experiments were reviewed and approved by the Pusan National University Institutional Animal Care and Use Committee and carried out in accordance with the provisions of the National Institutes of Health Guide for the Care and Use of Laboratory Animals (Approval Number PNU-2020-2574 and PNU-2020-2641).

Note that full information on the approval of the study protocol must also be provided in the manuscript.
